# Magnitude, Severity, and Associated Factors of Anemia among Under-Five Children Attending Hawassa University Teaching and Referral Hospital, Hawassa, Southern Ethiopia, 2016

**DOI:** 10.1155/2020/7580104

**Published:** 2020-08-07

**Authors:** Yeshimebet Gebereselassie, Mesganaw BirhanSelassie, Tadesse Menjetta, Jemal Alemu, Aster Tsegaye

**Affiliations:** ^1^Hawassa College of Health Science, Department of Medical Laboratory Science, Hawassa, Ethiopia; ^2^Hawassa University, College of Medicine and Health Science, Hawassa, Ethiopia; ^3^Department of Medical Laboratory Sciences, School of Allied Health Science, College of Health Science, Addis Ababa University, Addis Ababa, Ethiopia

## Abstract

**Background:**

Anemia is a widespread public health problem associated with increased risk of morbidity and mortality. Infants, under-5-year-old children, and pregnant women have greater susceptibility to anemia. The magnitude and associated risk factors for anemia vary in different settings. The study aimed to assess the magnitude, severity, and associated factors of anemia at Hawassa University Teaching and Referral hospital, Hawassa, southern Ethiopia.

**Methods:**

In a hospital-based cross-sectional study, a total of 422 under-five children were included. Sociodemographic data and other predisposing factors were collected by structured questionnaire. Venous blood samples were collected and analyzed for hemoglobin determination using a Cell-Dyn 1800 automated analyzer. Stool samples were collected and processed using direct wet mount and formol-ether concentration method to detect intestinal parasites. Data were entered and analyzed using SPSS version 20 statistical packages. Binary and multiple logistic regressions were computed to assess factors associated with anemia. *p* value less than 0.05 was taken as statistically significant.

**Result:**

The overall prevalence of anemia was found to be 41.7%. The mean hemoglobin level was 10.59 g/dl. Anemia was of mild, moderate, and severe type in 6.6%, 19%, and 16.1% of the children, respectively. Children in the age group 6–23 months (AOR = 2.04 (95% CI: 1.13, 3.69)), and mothers having no formal education (AOR = 1.73 (95% CI: 0.99, 3.02)) were identified as associated factors for anemia.

**Conclusion:**

The prevalence of anemia among the study subjects was 41.7% indicative of the fact that anemia is an important public health problem. It was associated with the child's age, residence, mother's education level, and intestinal parasite (*Ascaris lumbricoides*). It clearly indicates that there should be well integrated public health interventions to improve the health status that needs to be prioritized to prevent anemia among children under five years of age.

## 1. Background

World Health Organization defines anemia as a decrease in the concentration of circulating red blood cells or in the hemoglobin concentration and a related impaired capacity to transport oxygen. It is also defined as hemoglobin level below 11 mg/dl for children of 6–59 months [1].

Anemia is classified as microcytic, normocytic, or macrocytic, based on the mean corpuscular volume [2]. It can also be classified based on haemoglobinization of red cells as normochromic, describing normal staining of red cells, as seen when haemoglobinization is adequate, and hypochromic, describing pale staining of red cells, as seen when haemoglobinization is inadequate. Hypochromic cells show an increased area of central pallor [3].

Globally, anemia affects 1.62 billion people, which corresponds to 24.8% of the population. The highest prevalence is in preschool-age children which is 47.4%. World Health Organization regional estimates generated for preschool-age children and pregnant and nonpregnant women indicate that the highest proportion of individuals affected are in Africa (47.5–67.6%), while the greatest number is in South East Asia where 315 million individuals are affected [1].

World Health Organization showed that 818 million children under the age of five and women are affected by anemia, mainly in developing countries. About one million of them die every year [1]. In the developing world, 42% of children less than five years of age and 53% of children 5–14 years of age are anemic [4]. About 67.6% of under-five children in Africa are suffering from anemia indicating anemia as a severe health problem. In East Africa, it is estimated that three quarters of under-five children suffer from anemia [1]. Studies conducted in East Africa have shown that *P. falciparum* malaria and iron deficiency account for much of the anemia seen in young children [5].

In Ethiopia, according to the demographic and health survey findings in 2011, more than four out of ten under-five children (44%) were anemic. From these, about 21% of children were mildly anemic, 20% were moderately anemic, and 3% were severely anemic [6]. The etiology of anemia involves the interaction between multiple factors including nutritional deficiencies, genetic red blood cell disorders, and infectious diseases, particularly malaria and hookworm infections. Human immunodeficiency virus/acquired immunodeficiency syndrome should also increasingly be considered as a direct and indirect contributor to anemia in young age group [7–9].

Like other developing countries, the prevalence of intestinal parasites is widely spread in Ethiopia that can cause anemia in under-five children. Among the common intestinal protozoan parasites, *Giardia*, *Cryptosporidium*, and helminths such as *Ascaris* are widely distributed, where there is overcrowding, poor environmental sanitation, limited economic resources, and poor personal hygienic practice which are predisposing factors. Most of the intestinal parasites are more severe in children than adults, which is associated with malnutrition, growth retardation, and poor care for children. The case is worth in under-five-year-old children because of poor maternal hygiene, play habitats of children in the house in close proximity to one another that creates an appropriate condition for the transmission and spread of the disease [7].

A consequence of anemia may lead to impaired cognitive function, growth, and psychomotor development, especially in children. Infants, under-5-year-old children, and pregnant women have greater susceptibility to anemia because of their increased iron requirements due to rapid body growth and expansion of red blood cells [9–11]. The frequency of anemia in a population may be the most important single indicator of the overall health status of a population regarding malaria, geohelminths infection, and mal- or undernutrition [9].

Anemia in preschool children has a negative effect on cognition, motor development and growth, academic performance, immunity, and susceptibility to infections [12]. Despite these, detailed investigation of anemia in under-five children is limited in Ethiopia and existing data revealed variable magnitude. Therefore, this study is designed to assess the magnitude of anemia and its associated factors among under-5 children attending Hawassa University Teaching and Referral hospital in Hawassa, southern Ethiopia.

## 2. Methods

### 2.1. Study Area

The study was conducted at Hawassa University Teaching and Referral hospital, located in the city of Hawassa, southern Ethiopia. Hawassa city is found about 270 km south of the capital Addis Ababa.

### 2.2. Study Design

A cross-sectional hospital-based study was conducted to determine the magnitude of anemia and associated factors among under-5 children at Hawassa city, southern Ethiopia.

### 2.3. Study Subjects

All under-five children who were visiting the hospital during the study period and whose parents consented to participate in the study were included. Before data collection, written informed consent was obtained from each parent/caregiver of the study participants after explaining the purpose of the study.

### 2.4. Data Collection

Semistructured questionnaire was used to collect information such as patients' age, sex, residence, presence of malaria parasite, presence of intestinal parasites, mother's age, and mother's education. Data about the number of children less than 5 years were obtained from their parents/guardians. The tool was prepared after reviewing different related literatures inside and outside the country. The original questionnaire which was prepared in English language was translated into Amharic version and used by trained nurses for data collection. In addition, blood and stool samples for laboratory analyses were collected and analyzed by experienced laboratory professionals.

### 2.5. Sample Size and Sampling Technique

The minimum sample size required for analysis was calculated using the 95% confidence interval with 5% marginal error, using the formula *n* = *z*^2^*p* (1 − *p*)/*d*^2^, where *n* = sample size, *z* = statistic for a level of confidence (*z* = 1.96 at 95% CI), *p* = expected prevalence or proportion (*p* = 0.50), and *d* = precision (if 5%, *d* = 0.05), and the sample size was 384. Allowing 10% for nonresponse, the final sample size was 422. A convenient sampling technique was used to select under-five children who fulfilled inclusion criteria during the study period.

### 2.6. Blood Sample Collection

About 3 ml of venous blood samples were collected by strictly following standard operating procedure. The blood was processed for hemoglobin test.

### 2.7. Stool Specimen Collection and Processing

About 1 gm (pea size) fresh fecal specimen was collected using a labeled, clean, dry, leak-proof container [13]. The stool specimen was processed by the wet mount and formol-ether concentration technique.

### 2.8. Data Management and Statistical Analysis

All questionnaires were checked for completeness. Completed data were coded, entered, cleaned, and analyzed using Statistical Package for Social Sciences version 23. Descriptive statistics were used to summarize continuous variables and frequencies tables were computed to show the distribution of the sociodemographic and clinical characteristics of the patients. To assess associations between risk factors and anemia, bivariate and multivariate logistic regression analyses were used. Odds ratio with 95% confidence level was used to determine the strength of association. Statistical significance was declared at *p* value <0.05.

### 2.9. Classification of Anemia

The definition of anemia and classification of severity of anemia were determined based on the hemoglobin value in accordance with the value stated by the World Health Organization [14].

### 2.10. Ethical Considerations

Ethical clearance was obtained from the ethical review board of Addis Ababa University College of Health Science, School of Allied Health Science, Department of Medical Laboratory Sciences and Institutional Review Board of the College of Medicine and Health Science of Hawassa University. Before data collection, written informed consent was obtained from each parent/caregiver of the study participants after explaining the purpose of the study.

## 3. Results

### 3.1. Characteristics of Study Participants

A total of 422 under-five children participated in the study. Of the participants, 235 (55.7%) were males and 187 (44.3%) females. The age ranged from 6 to 59 months with mean age of 27.9 (±16.72) months. Regarding their age distribution, 174 (41.2%) were between 6 and 23 months, 98 (23.2%) were between 24 and 35 months, 75 (17.8%) were between 36 and 47 months, and 75 (17.8%) were between 48–59 months (Table 1).

### 3.2. Parental Characteristics

All of the parents/guardians were female with mean age of 28.8 ± 5.0 years. Regarding the educational status, 119 (28.2%) had attained secondary education and above, 112 (26.5%) were at primary level, and 191 (45.3%) had no formal education (Table 1).

### 3.3. Prevalence of Anemia

The overall prevalence of anemia as defined by hemoglobin levels lower than 11 g/dl was found to be 41.7% (176/422). Of them, 44.3% (78/176) were females and 55.7% (98/176) were males. The frequency of anemia declined with age; it was highest in the youngest age group of less than 2 years (48.9%) and lowest (34.7%) in the oldest age group.

Regarding the severity of anemia, among sampled children, 68 (16.1%) of them were severely anemic, whereas 80 (19%) were moderately anemic and 28 (6.6%) were mildly anemic (Table 2).

### 3.4. Factors Associated with Anemia

Regarding the result of intestinal parasites among study subjects, overall, 77 (18.2%) were infected with intestinal parasites. As shown in Table 3, *Ascaris lumbricoides* accounted for the highest 47 (11.1%) followed by *E. histolytica* 18 (4.3%), hookworm 17 (4.0%), and *G. lamblia* 3 (0.7%). The prevalence was 44 (18.7%) for male and 33 (17.6%) for female. It was high among the age group of 48–59 months (32%) compared to other age groups.

In bivariate (unadjusted) logistic regression analysis, statistically significant associated factors of anemia were children aged 6–23 months, rural residence, children whose mothers had no formal education, and infection with *Ascaris lumbricoides*. However, in multivariate (adjusted) logistic regression analysis, statistically significant associated factors of anemia were only children aged 6–23 months while children whose mothers had no formal education had a borderline statistical significance.

Children aged 6–23 months had two times odds of being anemic than those aged 48–59 months (AOR = 2.0 (95% CI: 1.13, 3.69); *p* value 0.017). The odds of being anemic among children whose mothers had no formal education were nearly two times more compared to those children whose mothers had secondary and above education level (AOR = 1.7 (95% CI: 1.0–3.0); *p* value 0.051). However, this finding was with borderline statistical significance in multivariate but was statistically significant in bivariate analysis (Table 4). In bivariate (unadjusted) logistic regression analysis, children living in rural area had 1.2 times odds of being compared to their urban counterparts (AOR = 1.5 (95% CI: 1.0–2.3); *p* value 0.047). Furthermore, in bivariate (unadjusted) logistic regression analysis, children who were infected with *Ascaris lumbricoides* had 2.5 times odds of being anemic than those who were not (AOR = 2.5 (95% CI: 1.3–4.7); *p* value 0.004).

## 4. Discussion

This study showed that the overall prevalence of anemia among under-five children was 41.7% and the mild, moderate, and severe anemia were 6.6%, 19%, and 16.1%, respectively. The prevalence of anemia in this study is comparable with findings in Bangladesh (40.9%) conducted in 2010 [15], North Kazakhstan (40.0%) [16], and national prevalence EDHS 2011 (44%) [6]. However, the finding is lower than that of estimated global anemia prevalence (47.4%) [1] and those reported from Ghana (78.4%) in 2014 [17], Cape Verde (51.8%) in 2014 [18], northern part of Ethiopia (50.3%) [19], Nigeria (70.5%) in 2012 [20], Tanzania (77.2%) in 2015 [21], and Kenya (71.8%) in 2005 [11]. The lower prevalence in this study might be due to the change made by the existing nutritional, public health interventions, easy accessibility of health information through health extension workers, and among other factors that may be included. But it is higher than the study conducted in Eastern Cuba in 2011 which is 26% [22], 28.8% from Kenya in 2014 [10], and 38.8% from Haiti 2013 [23]. The possible reason for these differences may be due to geographical and seasonal differences, interventions used, lifestyles, and socioeconomic status.

In this study, the magnitude of severe anemia among the study participants was 16.1%. This result is lower than the study conducted in Tanzania (27.7%) [21]. But it was higher than the findings in western China which documented 3.2% severe anemia [24]. In this study, the severity of anemia significantly associated with residence; children living in rural area were highly susceptible to severe anemia. A similar finding was reported in Tanzania [25].

In the present study, children who were less than 23 months were found more likely to be anemic. This finding is similar to studies conducted in southern Cameroon in 1998 [25], northern Ethiopia in 2014 [26], and Tanzania in 2015 [21]. Children of mothers with no formal education were more anemic than those with mothers having secondary and above level of education. Educated mothers are more conscious of their children's health and introducing scientifically proven feeding practices, which help to improve their children's nutritional status [27]. This result is comparable with studies conducted in northeast Ethiopia (2015) [28], Burma (2012) [29], Tanzania (2015) [21], Brazil (2013) [30], and India (2014) [12].

In this study, in bivariate logistic regression analysis, children who were infected with *Ascaris lumbricoides* infection were more likely to be anemic. This result was supported by reports from Nigeria [31] and Malaysia [32], but studies conducted in Indonesia [33] and Senegal [34] show no association between *Ascaris lumbricoides* infection and anemia.

## 5. Conclusion

The prevalence of anemia among the study subjects was 41.7% indicative of the fact that anemia is an important public health problem. It was associated with the child's age, residence, mother's education level, and intestinal parasite (*Ascaris lumbricoides)*. It indicates that there should be well integrated public health interventions to improve the health status that needs to be prioritized to prevent anemia among children under five years of age.

## Figures and Tables

**Table 1:** Sociodemographic characteristics of children aged 6–59 months and their parents/guardians attending Hawassa University Teaching and Referral hospital, Hawassa, southern Ethiopia, 2016.

Parameters	Frequency	Percentage
Age of child (in months)		
6–23	174	41.2
24–35	98	23.2
36–47	75	17.8
48–59	75	17.8

Sex of child		
Male	235	55.7
Female	187	44.3

Parents/guardians characteristics		
Residency		
Urban	284	67.3
Rural	138	32.7

Age group (in year)		
<30	241	57.1
≥30	181	42.9

Educational status		
No formal education	191	45.3
Primary	112	26.5
Secondary and above	119	28.2

Occupation		
Housewife	140	33.2
Employed	116	27.5
Small scale business	79	18.7
Farmer	80	19.0
Others	7	1.6

**Table 2:** Distribution of hemoglobin level among male and female under-5 children at Hawassa University Teaching and Referral hospital, Hawassa, southern Ethiopia, 2016.

Hgb value	Male (%)	Female (%)	Total (%)
<7 g/dl (severe)	40 (17.0)	28 (15.0)	68 (16.1)
7–9.9 g/dl (moderate)	40 (17.0)	40 (21.4)	80 (19.0)
10–10.9 g/dl (mild)	18 (7.7)	10 (5.3)	28 (6.6)
≥11 g/dl (nonanemic)	137 (58.3)	109 (58.3)	246 (58.3)

**Table 3:** Frequency of intestinal parasites among under-5 children in Hawassa University Teaching and Referral hospital, Hawassa, southern Ethiopia, 2016.

Species	Positive	Negative
	*N* (%)	*N* (%)
*Ascaris lumbricoides*	47 (11.1)	375 (88.9)
Hookworm	17 (4.0)	405 (96.0)
*Entamoeba histolytica*	18 (4.3)	404 (95.7)
*Giardia lamblia*	3 (0.7)	419 (99.3)

**Table 4:** Multivariate logistic regressions of selected variables associated with anemia among under-five children in Hawassa University Teaching and Referral hospital, southern Ethiopia, 2016.

Risk factor	Anemia	Unadjusted (COR)		Adjusted (AOR)	
	*N* (%)	OR (95% CI)	*p* value	OR (95% CI)	*p* value
Child age					
6–23	85 (48.9)	1.8 [1.0–3.1]	0.040	2.04 [1.13–3.69]	0.017
24–35	39 (39.8)	1.2 [0.7–2.3]	0.490	1.52 [0.78–2.91]	0.211
36–47	26 (34.7)	1 [0.5–1.9]	1.000	1.11 [0.55–2.24]	0.759
48–59	26 (34.7)	1		1	

Residence					
Urban	109 (38.4)	1		1	
Rural	67 (48.6)	1.5 [1.0–2.3]	0.047	1.24 [0.76–2.02]	0.378

Mother's education level					
No formal	62 (48.1)	1.67 [1.0–2.7]	0.033	1.74 [1.00–3.02]	0.051
Primary	57 (42.9)	1.4 [0.8–2.2]	0.207	1.43 [0.82–2.52	0.208
Secondary or above	57 (35.6)	1		1	

*A. lumbricoides*					
Yes	29 (61.7)	2.5 [1.3–4.7]	0.004	2.44 [0.93–6.46]	0.071
No	147 (39.2)	1		1	

## Data Availability

The datasets used and/or analyzed during the current study are available from the corresponding author on reasonable request.

## Additional Points

*Study Limitations.* In this study, only anemia was measured, the inclusion of other measures such as serum ferritin would help to measure the status of iron.
